# Disparities in cancer‐related healthcare among people with intellectual disabilities: A population‐based cohort study with health insurance claims data

**DOI:** 10.1002/cam4.3333

**Published:** 2020-07-25

**Authors:** Maarten Cuypers, Hilde Tobi, Cornelis A. A. Huijsmans, Lieke van Gerwen, Michiel ten Hove, Chris van Weel, Lambertus A. L. M. Kiemeney, Jenneken Naaldenberg, Geraline L. Leusink

**Affiliations:** ^1^ Department of Primary and Community Care Radboud Institute for Health Sciences Radboud University Medical Center Nijmegen The Netherlands; ^2^ Biometris Wageningen University & Research Wageningen The Netherlands; ^3^ Vektis Healthcare Information Center Zeist The Netherlands; ^4^ Department of Health Services Research and Policy Honorary Professor of Primary Health Care Research Australian National University Canberra Australia; ^5^ Department for Health Evidence Radboud Institute for Health Sciences Radboud University Medical Center Nijmegen The Netherlands

**Keywords:** early detection of cancer, healthcare disparities, hospital, intellectual disability, neoplasms, oncology service

## Abstract

**Background:**

Concerns have been raised about the accessibility and quality of cancer‐related care for people with intellectual disabilities (ID). However, there is limited insight into cancer incidence and the utilization of cancer care at the ID population level to inform targeted cancer control strategies. Therefore, we aimed to examine differences in the utilization of cancer‐related care between people with and without ID, identified through diagnostic codes on health insurance claims.

**Methods:**

In a population‐based cohort study, Dutch individuals of all ages who received residential care through the Chronic Care Act due to an ID (n = 65 183) and an age and sex‐matched sample of persons without ID (1:2 ratio), who were cancer‐free at enrollment in 2013 were followed through 2015. Incidence rates (IRs) of newly started cancer care and IR ratios (IRRs) with 95% CIs were used to compare groups. Separate analyses were performed per cancer type.

**Results:**

Individuals with ID received less cancer‐related care than individuals without (IRR = 0.64, 95% CI 0.62‐0.66). Differences increased with age and were larger for females than for males. Utilization of care for cancers within the national screening program (female breast, cervical, and colon cancer) was lower for people with ID compared to people without ID.

**Conclusion:**

Cancer may be underdiagnosed and/or undertreated in people with ID, or cancer is truly less prevalent in this population. In particular, the differences detected between males and females with ID, and the potential underutilization of national screening programs, require urgent follow‐up investigations.

## Graphical abstract

Individuals with an intellectual disability (ID) were given less cancer‐related health care than people without ID. This could indicate cancer is under‐diagnosed and/or under‐treated in people with ID.

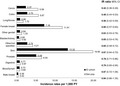

## 1 INTRODUCTION

Cancer care is well embedded in primary and community care but faces challenges when it comes to people with intellectual disabilities (ID). Individuals with ID constitute at least 1% of the population in Western countries and have significant impairments in intelligence and social functioning, developed before adulthood, often by a genetic cause.^1^, ^2^, ^3^, ^4^ Increasingly, people with ID live in community settings and rely on the same healthcare system as people in the general population.^5^ Difficulties in accessing healthcare, communication problems, and their dependence on others lead to significant health disparities and even elevated risks of premature mortality attributable to preventable and treatable causes, including cancer.^6^, ^7^

Concerns about the accessibility and quality of cancer‐related care for people with ID have been substantiated by studies indicating lower participation rates in cancer screening, detection of cancer at higher tumor stages, different distribution patterns across affected organs, and cancer‐related mortality at a younger age compared to non‐ID reference groups.^8^, ^9^, ^10^, ^11^, ^12^, ^13^, ^14^, ^15^ However, as these studies addressed only particular aspects of cancer control or related care, insight into the actual and recent cancer incidence within the ID population at large and the utilization of cancer‐related healthcare is still limited.^16^, ^17^

Quantification of the cancer burden in the ID population is challenged by two main issues: (a) studies often focus on small subgroups (e.g. people with a particular syndrome such as Down syndrome), and, consequently, these studies cannot be generalized to the population of everyone with ID; (b) there are, for a series of reasons, only a few countries with functional registers of people with ID.^13^, ^18^ People with ID are, therefore, difficult to identify in population‐based data, and they are often excluded in health surveys.^2^, ^3^, ^19^, ^20^, ^21^, ^22^, ^23^ Consequently, many studies have had to rely on convenience sampling, which may have resulted in biased outcomes, and other studies contained such small samples that they would have had insufficient power to detect statistically significant differences between ID subgroups and the general population.^16^, ^17^, ^24^, ^25^

The need for more information from well‐powered studies on cancer incidence and cancer‐related care in the entire ID population has been widely recognized.^22^, ^24^ Thus, we conducted a population‐based cohort study to compare the utilization of cancer‐related care between people with and without ID, identified through diagnostic codes on health insurance claims.

## 2 METHODS

### 2.1 Setting and design

A mandatory health insurance for a statutory insurance package is the basis of the Dutch national healthcare system, covering essentially all inhabitants.^26^ Care providers and health insurers within the healthcare system are regulated by the Dutch Healthcare Authority (NZa). After referral by a primary care physician (usually a GP), all inpatient and outpatient hospital care, including all cancer‐related care, is paid for by health insurers. As individuals with and without ID rely on the same insurance mechanism, their health insurance claims data provides information on the utilization of cancer‐related hospital care. The Vektis Healthcare Information Center routinely receives claims data from all Dutch health insurers, containing diagnostic codes (using the NZa classification table), date of care utilization, and a personal unique identifier to retrieve the patient's date of birth and sex. Furthermore, Vektis has access to the database containing data on individuals who are eligible to ID‐specific care services under the Dutch Chronic Care Act because of a registered ID diagnosis (DSM‐IV and WHO criteria).^4^ These ID services include all possible routine personal, daily, and social care to individuals with who cannot live independently, the vast majority with moderate to severe ID. The population under study range from individuals who can manage themselves with some residential support, to individuals who are immobile and require day‐long guidance and care. For the purposes of this research, Vektis representatives (CH, LvG, and MtH) created cohorts (see next section), ran queries, and aggregated data into ten‐year age groups to ensure patients' privacy, before sharing data with the research team. The study protocol was reviewed by the Radboud university medical center institutional Ethics Committee who passed a positive judgment (2017‐3921). We report our results in accordance with the STROBE statement.^27^

### 2.2 Cohorts

The ID cohort contained all Dutch individuals of all ages with a registered ID diagnosis in the Chronic Care Database, at any point in the four‐year period between 2012 and 2015, and who were alive on 31 January 2012. To generate a general population reference cohort, each individual from the ID cohort was randomly matched by age and sex to two individuals without an ID diagnosis and alive on 31 January 2012. The 1:2 ratio was chosen to allow for exact matching and robust comparisons without overpowering.^28^ Claims were retrieved from the health insurance claims database for the years 2012‐2015, based on the date of cancer care utilization, not the date on which the claim was submitted.

### 2.3 Outcome measure

The primary outcome measure is an insurance claim for hospital care for which an oncological diagnosis was reported as cause and for which care was utilized between 1 January 2012 and 31 December 2015. A reported cancer diagnosis was presumed to be new if the same diagnosis had not been reported on any claim in the previous year. Here, we assumed that, when cancer is detected, at least one insurance claim is expected in the following year as well, for treatment, surveillance, or follow‐up. Therefore, we used the first year (2012) to establish patients with ongoing cancer care and only report on individuals who newly started cancer care for the remaining three‐year follow‐up period (2013‐2015).

### 2.4 Statistical analyses

We assumed mortality to be distributed equally over each year and assigned 0.5 person‐years (PY) to individuals for the year in which they died. Other causes of loss‐to‐follow‐up (e.g. emigration, people becoming uninsured) were assumed to be small and equal for both cohorts. The three‐year period incidence rates (IRs per 1000 PY) and IR ratios (IRRs) of cancer‐related care were calculated.^29^ IRs and IRRs were also calculated per age group and sex stratum, and separate analyses were performed per cancer type. All estimates were presented with 95% confidence intervals (CIs). Analyses were conducted using SPSS (version 25.0) and Microsoft Excel (version 2016).

## 3 RESULTS

**FIGURE 1:**
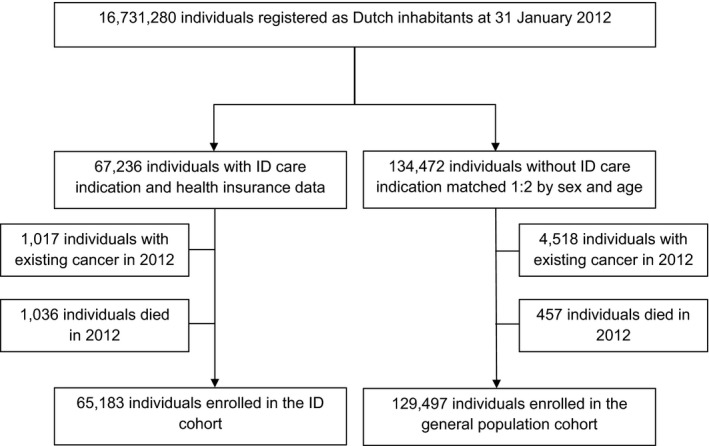
ID and general population cohort selection

Data were retrieved from the health insurance claims database for 67 236 individuals with ID, and 134 472 matched individuals from the general population. After individuals with prevalent cancer were excluded, 65 183 individuals with ID were enrolled in the ID cohort and 129 497 in the general population reference cohort (Figure 1). The cohorts were similar in terms of age and sex distribution (Table 1).

**TABLE 1:** Characteristics of ID and general population cohort

	Intellectual disability cohort N = 65 183	General population cohort N = 129 497
Mean age (SD)^a^	42.3 (17.8)	42.3 (17.8)
Age groups, N (%)^b^		
0‐9	666 (1.0%)	1333 (1.0%)
10‐19	5938 (9.1%)	11 906 (9.2%)
20‐29	13 717 (21.0%)	27 487 (21.2%)
30‐39	9050 (13.9%)	18 100 (14.0%)
40‐49	12 253 (18.8%)	24 445 (18.9%)
50‐59	12 096 (18.6%)	23 905 (18.5%)
60‐69	7616 (11.7%)	14 996 (11.6%)
70‐79	2994 (4.6%)	5618 (4.3%)
80‐89	796 (1.2%)	1618 (1.2%)
≥90	54 (0.1%)	113 (0.1%)
Sex, N (%)		
Male	36 927 (56.7%)	73 516 (56.8%)
Female	28 256 (43.3%)	55 981 (43.2%)
Cancer‐related care		
Person‐years at‐risk	190 533	386 145
Newly started cancer care	5513	17 485
Incidence rates	28.9 per 1000 PY	45.3 per 1000 PY
Incidence rate ratio [95% CI]	0.64 [0.62‐0.66], *P* < .001	

^a^ Age at recruitment (2012).

^b^ Distribution across age groups at start of follow‐up (2013).

### 3.1 Overall new cancer‐related care

**FIGURE 2:**
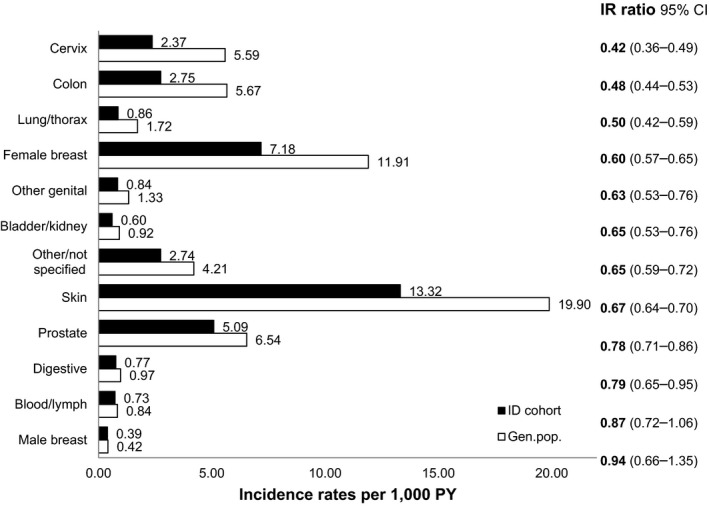
Incidence rates (IRs) and incident rate ratios (IRRs) per cancer type for ID and general population cohort, sorted by IRR

During the three‐year follow‐up, we found 5513 individuals who started cancer care with a new diagnosis in the ID cohort (IR = 28.9 per 1000 PY) compared to 17 485 individuals in the general population cohort (IR = 45.3 per 1000 PY), resulting in an overall IRR of 0.64 (95% CI 0.62‐0.66) (Table 1). Skin cancer was the most prevalent cancer type in both the ID cohort (IR = 13.32) and the general population cohort (IR = 19.90) (Figure 2). All types of cancer yielded estimated IRRs <1.0, although this was not statistically significant for male breast cancer and blood cancers/lymphomas (Figure 2). The lowest IRRs were found for lung cancer (IRR = 0.50; 95% CI 0.42‐0.59) and for cancer types with national screening programs: cervical (IRR = 0.42; 95% CI 0.36‐0.49), colon (IRR = 0.48; 95% CI 0.44‐0.53), and female breast (IRR = 0.60; 95% CI 0.57‐0.66) (Figure 2).

### 3.2 Sex and age subgroups

The overall IRR was lower for females with ID (IRR = 0.59; 95% CI 0.57‐0.62) than for males with ID (IRR = 0.69; 95% CI 0.66‐0.72; Table 2). Care related to cervical cancer yielded the smallest IRR for females (IRR = 0.42; 95% CI 0.36‐0.49); for males, IRRs were lowest for both colon (IRR = 0.48; 95% CI 0.43‐0.54) and lung cancer (IRR = 0.48; 95% CI 0.38‐0.59).

**TABLE 2:** Incidence rates (IRs) and incident rate ratios (IRRs) per cancer type and age group for ID and general population cohort, stratified by sex

	Females					Males				
	ID cohort IR	General population cohort‐IR	IRR	95% CI	*P*‐value	ID cohort IR	General population cohort‐IR	IRR	95% CI	*P*‐value
All cancers, all ages	33.56	56.65	0.59	0.57‐0.62	**<**.**0001**	25.40	36.61	0.69	0.66‐0.72	**<**.**0001**
Cancers with screening										
Female breast	7.18	11.81	0.60	0.55‐0.66	**<**.**0001**	‐	‐	‐	‐	
Cervical	2.37	5.59	0.42	0.36‐0.49	**<**.**0001**	‐	‐	‐	‐	
Colon	2.58	5.28	0.49	0.42‐0.57	**<**.**0001**	2.87	5.96	0.48	0.43‐0.54	**<**.**0001**
Cancers without screening										
Skin	14.56	23.85	0.61	0.57‐0.65	**<**.**0001**	12.37	16.86	0.73	0.69‐0.78	**<.0001**
Other/not specified	3.10	5.19	0.60	0.52‐0.68	**<**.**0001**	2.47	3.45	0.72	0.62‐0.82	**<**.**0001**
Prostate	‐	‐	‐	‐		5.09	6.54	0.78	0.71‐0.86	**<**.**0001**
Lung/thorax	0.80	1.47	0.54	0.42‐0.71	**<**.**0001**	0.91	1.92	0.48	0.38‐0.59	**<**.**0001**
Other genital	1.57	2.82	0.56	0.46‐0.67	**<**.**0001**	0.27	0.16	1.62	0.99‐2.63	.06
Bladder/kidney	0.57	0.60	0.95	0.67‐1.33	.76	0.62	1.17	0.53	0.41‐0.70	**<.0001**
Digestive	0.95	0.91	1.04	0.80‐1.36	.76	0.62	1.02	0.61	0.46‐0.80	.**0003**
Blood/lymph nodes	0.66	0.83	0.79	0.58‐1.08	.13	0.79	0.84	0.94	0.73‐1.21	.62
Male breast	‐	‐	‐	‐		0.39	0.42	0.94	0.65‐1.35	.76
Age groups										
0‐9	7.82	6.13	1.28	0.42‐3.90	.67	4.50	7.45	0.60	0.24‐1.50	.28
10‐19	20.01	21.07	0.95	0.78‐1.16	.62	13.02	11.10	1.17	0.95‐1.45	.14
20‐29	27.84	32.26	0.86	0.78‐0.96	.**007**	15.52	11.48	1.35	1.18‐1.54	**<**.**0001**
30‐39	30.67	46.33	0.66	0.59‐0.75	**<**.**0001**	14.11	18.51	0.76	0.65‐0.89	.**0005**
40‐49	34.01	63.99	0.53	0.48‐0.58	**<**.**0001**	21.18	25.06	0.85	0.76‐0.95	.**0005**
50‐59	37.06	68.21	0.54	0.50‐0.60	**<**.**0001**	32.35	46.17	0.70	0.64‐0.77	**<**.**0001**
60‐69	45.45	88.28	0.51	0.46‐0.57	**<**.**0001**	54.38	99.87	0.54	0.50‐0.59	**<**.**0001**
70‐79	47.42	98.61	0.48	0.41‐0.56	**<**.**0001**	67.41	138.40	0.49	0.43‐0.56	**<**.**0001**
80‐89	42.61	73.85	0.58	0.43‐0.78	.**0003**	46.10	143.75	0.32	0.22‐0.46	**<**.**0001**
≥90	10.81	67.51	0.16	0.02‐1.21	.04	52.63	86.96	0.61	0.06‐5.82	.73

Note

Bold values indicate *P* < .01. Incidence rates per 1000 PY.

Among the youngest age groups (<20 years), no statistically significant differences in the risk of starting cancer‐related care were found between the two cohorts. With increasing age, IRRs declined (Table 2). IRRs were smallest for females between 70 and 79 years of age (IRR = 0.48; 95% CI 0.41‐0.56) and males between 80 and 89 years of age (IRR = 0.32; 95% CI 0.22‐0.46).

## 4 DISCUSSION

This study is the first to have detected an association between ID and a lower utilization rate of cancer‐related healthcare, using population‐based data from a healthcare system that aims to provide the same standards of care to people with and without ID. Our findings show that both females and the older age groups with ID received less cancer‐related healthcare than comparable individuals without ID. The largest absolute difference in IRs was found for skin cancer. Cancers for which national screening programs are implemented, as well as lung cancer, showed the largest relative differences between people with and without ID (IRRs).

While people with ID generally are higher health care users,^30^, ^31^ our analyses do not show this for the utilization of cancer care. Reasons for the lower utilization of cancer care in this population could be twofold: (a) the incidence of some cancer types is truly lower in the ID population than in the general population, and (b) some cancer types are underdiagnosed in individuals with ID or are undertreated after diagnosis. With respect to a truly lower cancer incidence in the ID population, more research is required into the genetic relation between specific ID syndromes and tumor growth propensity. Currently, this relation has been well documented only for Down syndrome, with a lower incidence of solid tumors but increased risks for leukemia.^32^, ^33^, ^34^, ^35^ The study of both underdiagnosis and undertreatment requires the integration of epidemiological, clinical, patient‐related, and ethical perspectives. Given the current results, future studies should investigate tumor stage at diagnosis and cancer‐related mortality, through a similar large comparative study.^11^ Research on knowledge and awareness in patients and their relatives of cancer‐specific risk factors could help to understand, and eventually to avoid, delays in seeking help and warning signals being missed. Such so called blocking mechanisms have been previously detected among ID staff and caregivers.^36^, ^37^, ^38^ Indicative of cancer possibly going unnoticed is our finding that blood and lymphatic cancers, which typically present clearly recognizable and visible symptoms at an early stage, did not show differences in received care, in contrast to most other cancer types that may start with less obvious symptoms. In these situations with patients from the general population, GPs often act on the basis of a premonition that something is wrong, generated by experience and intuition, which may require additional training to also be applied accurately when dealing with people with ID.^39^ Furthermore, ethical perspectives are needed to understand and provide shared decision‐making on the different choice in cancer treatment and other cancer‐related care, in relation to quality of life.^40^, ^41^

Screening programs for several cancer types have been introduced to give access to (early) diagnosis and are and have had an important role in public cancer control since. Nonetheless, there are many concerns about their functionality for people with ID. These concerns relate to lack of knowledge among people with ID and staff about cancer risk factors,^9^, ^14^ screening benefits,^12^, ^14^ and procedures,^42^, ^43^ and about physical barriers to access the screening location,^10^, ^14^ and, reluctance, and behavioral and physical barriers to undergo procedures.^8^, ^10^, ^42^, ^43^ The putative lack of functionality and subsequent diagnostic and treatment delays may at least partly explain the relatively low rates for female breast, cervical, and colon cancer care among individuals with ID. Large‐scale studies on possible ways to better engage people with ID in screening programs are, therefore, essential.

The rates of received cancer care presented in this study were higher than expected based on actual cancer diagnoses as given in literature.^44^ In part, this may be caused by the use of cancer diagnoses according to the NZa classification, which contains more diagnosis options than the usual International Classification of Diseases (ICD‐10).^45^ Because the ICD‐10 classes partially overlap with NZa codes, it is impossible to categorize NZa classes into ICD‐10 classes.

This study included an entire ID population that availed of the provisions of the Chronic Care Act with respect to residential ID care. As the Chronic Care Act is the only system available for this type of care, coverage of the population with moderate to severe ID will be almost 100%. The coverage of the population with mild or borderline ID is less good, as substantial part of them might not use any residential care service. Disabilities and impairments are expected to be less severe among those who remained unidentified, but exposures to risk factors (e.g. smoking, unsafe sex) could be higher and support networks less extended and knowledgeable than in the ID population that did receive ID‐related care.^46^ This makes the association found in the current study between having an ID and receiving cancer care relevant to this group as well. Specific cancer sites (e.g. esophagus, stomach, liver, gallbladder, and pancreas) had to be aggregated into a broader group (e.g. digestive cancers) to guarantee anonymity and avoid any risk of disclosure, even though the present study is population‐based. As the primary function of the data requires some personal identifiers only (i.e. sex and date of birth), these variables were used to match individuals from both cohorts. No additional information on possible confounding variables was available for matching. As severity of ID was not available either, only age and sex group‐specific IRs could be computed.

## 5 CONCLUSION

People with ID receive less cancer‐related care compared to people without ID. Further research is needed to investigate whether there is a true lower cancer incidence among this group or possible trajectories of underdiagnosis and subsequent undertreatment. It is of great importance that care providers can recognize, understand, and interpret symptoms that may be specific to patients with ID. Our study demonstrates the importance of population‐based studies on people with ID, as the patterns that we have detected would have otherwise remained undiscovered.

## CONFLICT OF INTEREST

Authors declare to have no conflicts of interest.

## AUTHOR CONTRIBUTIONS

GL, JN, LvG, MtH, and CH designed the study. CH, LvG, and MtH were responsible for the data, including data extraction from the Vektis databases, and aggregation. HT and MC conducted the statistical analyses and interpreted the data. MC, HT, and GL wrote the draft of the manuscript. CvW, LK, and JN contributed to interpretation of the findings. The report was edited by all authors, and all authors have approved the final version.

## Data Availability

This study used existing data that was obtained upon request from Vektis Healthcare Information Center and is subject to license restrictions. Procedures to request data can be found at www.vektis.nl. Aggregated data supporting the findings of this study are available from the corresponding author (MC) upon request.
